# Repetitive Sinus-Related Symptoms May Accelerate the Progression of Chronic Maxillary Atelectasis

**DOI:** 10.1155/2017/4296195

**Published:** 2017-07-03

**Authors:** Shu Kikuta, Kyohei Horikiri, Kaori Kanaya, Ryoji Kagoya, Kenji Kondo, Tatsuya Yamasoba

**Affiliations:** Department of Otolaryngology, Graduate School of Medicine, University of Tokyo, 7-3-1 Hongo, Bunkyo-ku, Tokyo 113-8655, Japan

## Abstract

Chronic maxillary atelectasis (CMA) is characterized by a progressive decrease in maxillary sinus volume. The factors that promote the stage progression of CMA remain poorly understood. Here, we describe the time course of anatomical changes in a 40-year-old woman with stage II CMA that progressed to stage III disease. She did not show stage progression until she started to develop repetitive sinus-related symptoms. The stage progression was characterized by ocular symptoms. The repetitive inflammatory episodes may have increased the negative pressure in the affected sinus and weakened the bone walls, thereby promoting stage progression. Thus, a history of repetitive sinus-related symptoms may be a risk factor for stage progression in CMA.

## 1. Introduction

Chronic maxillary atelectasis (CMA) is characterized by a persistent and progressive decrease in the maxillary sinus volume and occlusion of the infundibulum as a result of inward bowing of the antral walls [1–4]. CMA is categorized into three stages on the basis of the degree of sinus wall deformation. Stage I is characterized by a lateralized maxillary fontanel (membranous deformity); stage II is defined as inward bowing of one or more of the osseous walls (bone deformity); and stage III is characterized by enophthalmos, hypoglobus, and/or midfacial deformity (clinical deformity) [1, 5]. Although several authors have previously reported cases of patients with CMA [2, 6–9], they only described the condition at one time point. We speculated that if we followed CMA patients as they progressed through the various stages of CMA, we might be able to identify factors that predict stage progression. We report here the case of a patient with stage II CMA who did not exhibit progression until she developed repetitive sinus-related symptoms. This development might be associated with the progression of the disease to stage III CMA. Our observations suggest that a history of repetitive sinus-related symptoms may be a risk factor for stage progression in CMA.

## 2. Case Presentation

**Figure 1:**
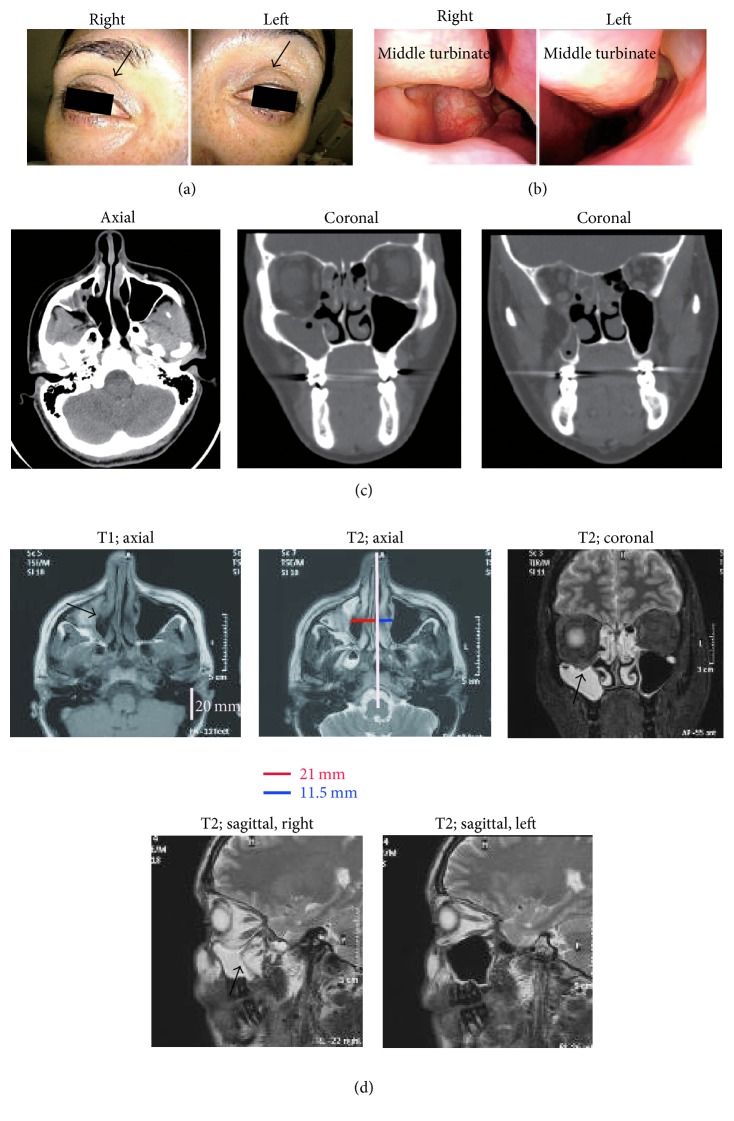
Summary of the clinical examination at the time the patient presented at our hospital. (a) Facial appearance: the arrows in the right and left pictures indicate the superior sulcus. The right superior sulcus appears to be deeper than the left superior sulcus. (b) Endoscopic findings: the right middle meatus is more enlarged than the left middle meatus. (c) Axial and coronal views on computed tomography. (d) T1 and T2 weighted images on magnetic resonance imaging. The T1 axial image shows that the right medial wall of the maxillary sinus (shown by the arrow) deviates laterally. The T2 coronal image indicates inferior bowing of the inferior wall of the orbit (shown by the arrow). The T2 sagittal images show prominent deviation of the posterior wall in the right maxillary sinus (shown by the arrow in the right image) relative to the structure in the left maxillary sinus (left image). The red and blue lines in the T2 axial image indicate the distance from the center line to the most deviated medial wall of the maxillary sinus.

A 40-year-old Hungarian woman suddenly noticed upgaze diplopia and right cheek compression when she woke up in the morning. Shortly thereafter, she consulted an ophthalmologist and then an otolaryngologist in another hospital. The investigations suggested that she had a carcinoma in the pterygopalatine fossa on the right side. Therefore, the otolaryngologist referred the patient to our institution for further examination. Examination of the facial appearance of the patient indicated more deepening of the right upper eyelid sulcus than the left eyelid sulcus (Figure 1(a)). An endoscopic examination showed that the right uncinate process could not be clearly detected and seemed to adhere to the medial wall of the maxillary sinus (Figure 1(b)). Computed tomography (CT) imaging then revealed inferior bowing of the floor of the orbit into the right maxillary sinus, lateral drifting of the right uncinate process into close contact with the floor of the orbit, and partial opacification of the maxillary sinus and anterior ethmoidal cells (Figure 1(c)). Magnetic resonance imaging (MRI) findings confirmed prolapse of the inferior inward retraction of the posterior, lateral, and medial walls of the maxillary sinus on the right side (Figure 1(d)). The patient had not experienced any trauma and did not have any endocrinological problems, developmental anomalies, and/or systemic diseases such as Wegener granulomatosis, orbital metastasis, osteomyelitis, progressive lipodystrophy, or facial hemiatrophy. The possibility of carcinoma in the pterygopalatine fossa was excluded by a FDG-positron emission tomography study. On the basis of these findings, the patient was diagnosed with stage III CMA.

**Figure 2:**
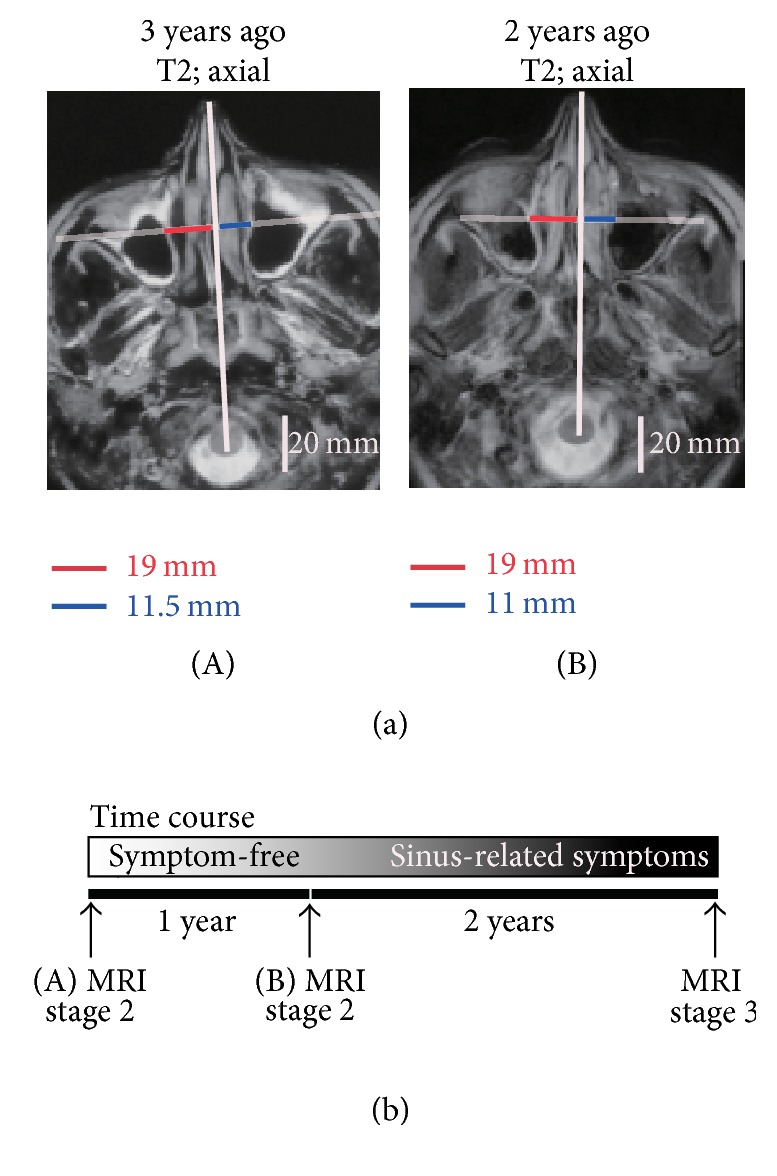
Magnetic resonance images taken 3 and 2 years before the development of ocular manifestations. (a) Magnetic resonance image (MRI) findings: (A) the T2 axial view of MRI 3 years before the ocular presentation; (B) the T2 axial view of MRI 2 years before the ocular presentation. Both images show that the medial wall of the right maxillary sinus is deviated compared to the medial wall of the left maxillary sinus. Similar anatomical changes are observed. Thus, 3 and 2 years before ocular manifestations appeared, the distances from the center line to the most deviated medial wall of the right maxillary sinus were 19 and 19 mm (red lines), respectively. By contrast, the distances from the center line to the most deviated medial wall of the left (unaffected) maxillary sinus were 11.5 and 11 mm (blue lines), respectively. Both situations indicate stage II disease. (b) Time course of the CMA patient. The first MRI was performed 3 years before ocular symptom presentation (A). The second MRI was performed 2 years before ocular symptom presentation (B). The patient did not present with any sinusitis-related symptoms before or at the first (A) and the second (B) MRI. However, in the 2 years following the second MRI, the patient frequently presented with sinus-related symptoms. At the end of that period, the patient was diagnosed with stage III disease.

Three years before the onset of the upgaze diplopia and right cheek compression, the patient had undergone brain MRI screening for a lacunar infarction. She had not previously presented with symptoms related to the nose or sinuses. When we reviewed these MRI images, we observed mucosal hyperplasia of the maxillary sinus and deviation of the medial wall of the maxillary sinus on the right side (as compared to the contralateral unaffected side). Thus, we retrospectively diagnosed the patient with stage II CMA. As shown by Figure 2(a), at that time point, the distance from the center line (defined as the line from the center of the midbrain to the nose tip) to the most deviated medial wall of the maxillary sinus was 19 mm on the right side (red line in (A)) and 11.5 mm on the left side (blue line in (A)).

Two years before the clinical onset of CMA, the patient underwent follow-up MR imaging. As shown by Figure 2(a), retrieval and retrospective examination of these images indicated that the anatomical deformity in the right maxillary sinus had not progressed (the red and blue lines in (B) indicated distances from the center line of 19 and 11 mm, resp.).

In the 2 years after the second MRI, the patient started complaining of repeated sinus-related symptoms such as cheek pain or pressure on both sides and anterior purulent nasal discharge once every 1 or 2 months (Figure 2(b)). She frequently consulted private Ear, Nose, and Throat Clinics to address these issues but was told it was due to sinusitis. At each visit, the patient was prescribed with a week-long course of antibiotics. Two years after the second MRI, the patient developed the ophthalmological symptoms, was referred to our hospital, and was diagnosed with stage III CMA (Figure 2(b)).

To treat the ocular symptoms, we used endoscopic sinus surgery (ESS) to eliminate the negative pressure within the maxillary sinus. We removed the laterally drifted uncinate process that was in close contact with the floor of the orbit and observed mucosal hypertrophy of the maxillary sinus. Pathology of the mucosae in the maxillary sinus revealed infiltration with inflammatory cells. After the treatment, the ocular symptoms of the patients disappeared rapidly.

**Figure 3:**
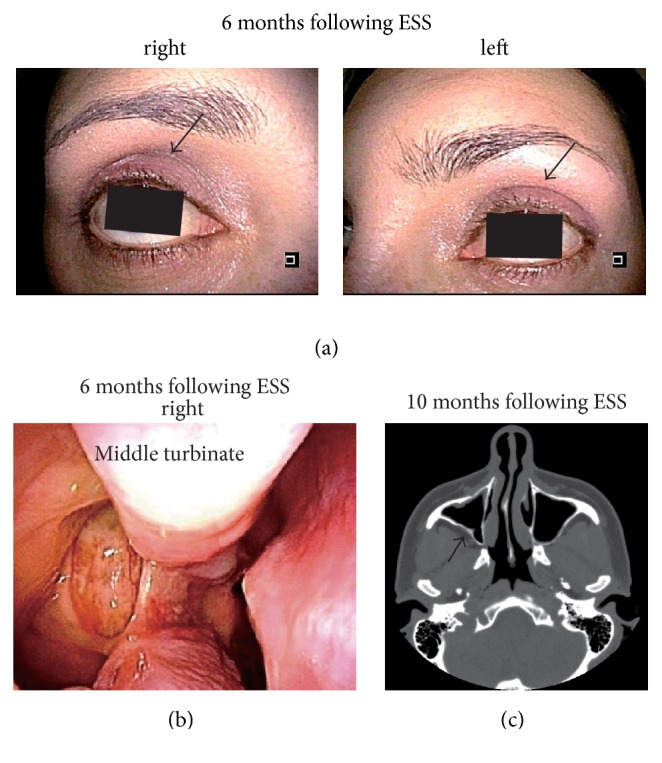
Summary of the clinical examination after the endoscopic sinus surgery. (a) Facial appearance 6 months after the endoscopic sinus surgery (ESS). The arrows in the right and left pictures show the superior sulcus. The deepening of the right superior sulcus that was observed before the sinus surgery appeared to have been eliminated by the ESS. (b) Endoscopic findings 6 months after the ESS. The right uncinate process was removed and recurrence of the deformity was not observed. (c) Computed tomography findings 10 months after the ESS. The deviation of the posterior wall in the right maxillary sinus (shown by the arrow) seems to have been eliminated by the ESS.

After surgery, we regularly followed the patient by endoscopy and CT imaging for more than 1 year. Six months after the ESS, right enophthalmos seemed to be equivalent to that on the affected side (Figure 3(a), arrow) and endoscopy showed that there was no recurrence of the nasal deformity (Figure 3(b)). Ten months after the ESS, CT imaging showed that the anatomical deformity of the posterior wall in the maxillary sinus had been repaired (Figure 3(c), arrow).

## 3. Discussion

We were able to observe the anatomical changes in our CMA patient as she progressed from stage II to stage III CMA over a period of 3 years. The progression of the condition appeared to be associated with the development of repeated sinus-related symptoms over 2 years. These symptoms preceded the development of the ocular symptoms that led to the diagnosis of stage III CMA. These observations suggest that a history of repetitive sinus-related symptoms may indicate progression of the anatomical deformity in CMA. As described below, these symptoms may be indicative of processes that promote CMA progression.

Occluded maxillary infundibulum produces an enclosed hypoventilated environment in the maxillary sinus [10, 11]. This enclosed cavity also induces air reabsorption within the affected sinus, which creates additional negative pressure. This leads to the eventual collapse of the maxillary sinus [12]. We speculate that the development of negative pressure in the affected sinus may also be further promoted by bacteria-induced inflammation of the mucosae in the occluded maxillary sinus, which enriches the capillary network of the sinus mucosae and increases the absorption of respiratory gases [13–15]. Moreover, repeated severe inflammatory changes in the mucosa may also induce immunological bone catabolism that causes thinning and demineralization of the bony wall [7]. Thus, we propose that a history of repetitive severe sinus inflammation that is accompanied by nasal symptoms may reflect promotion of negative pressure within the maxillary sinus and inflammation that weakens the bony wall. These processes ultimately cause the bony wall to bow inwardly, thus leading to stage progression in the CMA patient. We cannot rule out the possibility that negative pressure on its own is the key cause of stage progression in CMA. Nevertheless, our case suggests that the repetitive severe sinus inflammation may further facilitate the pathological consequences of the negative pressure within the maxillary sinus and therefore promote the collapse of the vulnerable bony wall.

It has been reported that some patients in the advanced stage have persistent ocular complaints after the negative pressure is removed by sinus surgery [16, 17]. This suggests that it may be necessary to remove the negative pressure while the CMA is at an early stage. This approach is further supported by our case, which suggests that patients who frequently experience sinus-related symptoms might be required to undergo sinus surgery to prevent further stage progression.

## 4. Conclusion

Our CMA patient had a history of repetitive sinus-related symptoms for 2 years before clinical and MRI evidence indicated that the CMA had progressed from stage II to stage III. Thus, a history of repetitive severe inflammation that is accompanied with sinus-related symptoms such as cheek pain or pressure and purulent nasal discharge may associate with stage progression of CMA. Early sinus surgery may prevent CMA stage progression and could be required if the patient frequently experiences sinus-related symptoms.
